# The Prevalence and Compliance of Health Claims Used in the Labelling and Information for Prepacked Foods within Great Britain

**DOI:** 10.3390/foods13040539

**Published:** 2024-02-09

**Authors:** Emma Coates, Kristina Pentieva, Hans Verhagen

**Affiliations:** 1 School of Biomedical Sciences, Faculty of Life and Health Sciences, Ulster University, Coleraine BT52 1SA, UK; emma@nualtra.co.uk (E.C.); k.pentieva@ulster.ac.uk (K.P.); 2 National Food Institute, Technical University of Denmark, Kemitorvet 201, 2800 Kgs. Lyngby, Denmark; 3 Food Safety & Nutrition Consultancy, 3703 EE Zeist, The Netherlands

**Keywords:** health claims, foods, compliance, Regulation 1924/2006

## Abstract

In the EU and Great Britain (GB), all health claims (HCs) on food must be authorised before use and should comply with Regulation 1924/2006. In GB, all HCs, authorised or not, are listed in the Great Britain Nutrition and Health Claims Register. This study reviews the prevalence and compliance of HCs on prepacked foods sold within three GB supermarkets and via their grocery shopping websites. In June 2023, food labels and online product information of 440 products were evaluated across three food categories—dairy and dairy alternatives; fruit juices, fruit juice drinks and fruit smoothies; and teas and infusions. In store, 26.3% of products carried an HC and 28.3% online. The prevalence of HCs was higher when compared with data from 2016. Overall compliance was high, in store (94.3%) and online (90.0%), with no statistically significant difference in overall HC compliance between in store and online products (*p* = 0.724). The HC violations observed in the present study were due to non-compliant wording of HCs or use of non-authorised HCs. This study demonstrates changes in the HC landscape and the need for continued monitoring of the prevalence and compliance of HCs as consumer trends alter.

## 1. Introduction

Labelling and advertisements are places for consumers to access information for the nutritional content of a food and/or its health benefits and may play a key role in giving consumers the information to make healthier dietary choices [1].

Health claims (HCs) are voluntary information included on food labels in order to inform the consumer that there is a relationship between health and consuming a food, a food category or one of its components [2]. In 2006, Regulation 1924/2006 on nutrition and health claims on food (NHCR) [2] was adopted in the EU. The use of nutrition and health claims (NHCs) is laid out in this regulation. All HCs must be scientifically substantiated, with evidence which demonstrates that there is a causal link between the intake of the food or one of its components and the health benefits described in the claim [3]. Nutrition claims (NCs) differ from HCs, in that they relate to the nutritional content of a product, for example, low fat or high protein. NCs are voluntary information, which states, suggests or implies that a food has specific benefits due to the following: (a) the calorific value it provides, does not provide, or provides at a reduced or increased rate; or (b) the nutrients or other substances it contains, does not contain, or contains in reduced or increased proportions [2].

The NHCR aims to ensure that consumers are protected against bogus NHCs and offers fair competition to food manufacturers whilst promoting and protecting innovation in the EU food sector. Since its adoption in the EU, over 250 HCs have been approved. These are listed on the EU Health Claims Register (EUHCR) [4]. Within GB, since Brexit, HCs continue to be regulated by Regulation (EC) No 1924/2006 (NHCR) as it has been retained in law. HCs must be authorised for use by the UK Government and the devolved nations’ administrations, following scientific advice and risk assessments via the UK Nutrition and Health Claims Committee [5]. In GB, many of the HCs currently in use are a legacy of retained EU authorisations following Brexit. The Great Britain Nutrition and Health Claims Register (GBNHCR) [6] provides a list of all authorised or non-authorised HCs. The use of authorised HCs must meet stipulated caveats, which are published with HC authorisation. The Department of Health and Social Care (DHSC) provides guidance on the compliance of HCs in accordance with the NHCR [7]. In GB, compliance of HCs is monitored by the Advertising Standards Authority (ASA), which encourages self-regulation. National Trading Standards (NTS) provide legal enforcement where ASA intervention has not resolved the inappropriate or inaccurate use of HCs [8]. 

In addition to the EUHCR and GBNHCR, there is also an Article 13.1 ‘on hold’ claims list for botanicals [9]. Botanicals and their derivatives are typically taken from plants, algae, fungi or lichens [10]. Despite being widely available and there often being a long history of use, there are safety and quality concerns regarding their use. The NHCR requires any HCs to be scientifically substantiated and preauthorised [11]. When the NHCR was adopted in 2006, it became apparent that the substantiation of botanicals and other ingredients was complex [12], and there were challenges to meet the requirements of the regulation. Following the adoption of the NHCR [2], over 2000 HCs for botanicals have been submitted to EFSA. However, following the publication of negative scientific opinions regarding botanical HCs, all HCs for botanicals were placed ‘on hold’ by the EC [11]. All claims that are not authorised, on hold or under consideration are prohibited [12].

Over the last decade, research has shown that HCs are present on between 10.5 and 25% of foods [13,14,15,16,17]. Kaur et al. (2016) [15] evaluated randomly selected foods across five European countries and, of the sample foods, 11% carried an HC. Offe et al. (2022) [17] assessed the prevalence of HCs on labelled foods available in Ireland and found that the use of HCs decreased from 21.6% in 2009 to 10.5% in 2021. The study revisited earlier data also collected in Ireland by Lalor et al. (2009) [18] and compared HCs on similar products. The decrease in HC prevalence could be attributed to changes in consumer habits, but the introduction of the NHCR shortly before the study by Lalor et al. (2009) may have had a greater impact. 

Lalor et al. (2009) [18] included a variety of dairy products, including milk, butter and spreads, cheese, yogurt and yogurt drinks, and other dairy. Over 30% of the milk, butter and spreads, and yogurts and yogurt drink products included in the study carried an HC. Yogurts and yogurt drink products carried the highest percentage of HCs at 50%. Teas were also included by Lalor et al. (2009) [18], of which 24% held an HC. In addition, fruit juices and smoothie products were also reviewed, revealing that 29% included an HC. Similar to Lalor et al. (2009) [18], Offe et al. (2022) [17] included yogurts and yogurt drinks as well as fruit juices and smoothies. HC prevalence remained high in both food categories, with 17.9% of yogurts and yogurt drink products and 31.8% of fruit juices and smoothies bearing an HC. The multicentred EU study by Hieke et al. (2016) [19] also included dairy products, and 28% held an HC. HC compliance was not included in any of the studies.

Research into the compliance of HCs in relation to the NHCR is limited. Studies conducted within several other European countries have shown a high prevalence of unauthorised or non-complaint HCs in use across a wide range of food categories, including teas and infusions [20], food supplements [21,22], sports supplements [23,24], meal replacement bars [25] and infant and child nutrition [26,27]. The data available within these studies demonstrate poor HC compliance, regardless of food category. Bonaccorsi et al. (2018) [28] evaluated the compliance of HCs present on the labels of various foods available in Italian supermarkets. Milk and fermented milk/yogurt products were included in the study, of which 62.5% of products were fully compliant with the NHCR. Kerrigan (2020) [20] found very poor compliance of HCs used on green/herbal teas sold via shops in Dublin, Ireland. The study concluded that 100% of HCs in use on the products that were reviewed were unauthorised and, therefore, non-compliant. Other violations of the NHCR highlighted by the data currently available include incomplete supporting HC information and failure to meet compositional standards or labelling requirements [20,25,26]. Dominguez et al. (2021) [21] demonstrated that there was more deceptive use of HCs via digital platforms than in physical settings when reviewing food supplements for pregnant women. However, more research is required to investigate this further in other food categories and within GB. For example, there are no HC compliance data available regarding fruit juices and fruit smoothies. The limited data regarding the NHCR compliance of HCs make it challenging to fully evaluate the extent of the issue. This also highlights that data are required to demonstrate where the problems lie and to support policymakers in regulating HCs, thus protecting consumers from misleading information.

The main aim of this study was to examine the compliance of HCs being used on prepacked foods, which are sold within GB supermarket stores and via their grocery shopping websites. In addition, the prevalence of HCs in store and online, the types of HCs being used and the food components (FCs), for example, a specific nutrient or ingredient, being used within the HCs were explored.

## 2. Materials and Methods

### 2.1. Study Samples

In this study, three food categories were investigated—dairy and dairy alternatives (D+DAs), fruit juices, fruit juice drinks and fruit smoothies (FJ,FJD+FS), and teas and infusions (T+Is). The product categories selected within this study are amongst those most commonly consumed according to Lalor et al. (2009) [18]; therefore, they were considered to be important categories to investigate as consumers are routinely exposed to them. The food categories within this study were also selected to reflect similar foods and categories included in previous studies conducted by Hieke et al. (2016) [19], Lalor et al. (2009) [18], Offe et al. (2022) [17], Bonaccorsi et al. (2018) [28] and Kerrigan (2020) [20], offering a range of data with which to compare the outcome of the study data. This study did not include all of the food categories that were investigated in larger studies by Hieke et al. (2016) [19], Lalor et al. (2009) [18] and Offe et al. (2022) [17] due to researcher resource limitations and time restraints. Chilled and ambient products were included in the D+DA and FJ,FJD+FS categories (see Table 1).

The aim was to evaluate approximately 400 products across the three food categories via three different grocery retailer stores and their grocery shopping websites. No statistical power calculation was made as this was a unique study, which evaluates in-store and online HC compliance. Whereas studies by Offe et al. (2022) [17] and Hieke et al. (2016) [19] included larger overall sample sizes, for comparable food categories, they included similar, yet slightly smaller, sample sizes. Three national supermarkets were included in the study (names not disclosed for privacy reasons). The retailers were selected to reflect supermarkets most frequently used by GB consumers [29,30]. Three other major retailers were considered but they were excluded due to limited product range and lack of permission to access the supermarket stores.

### 2.2. Data Collection

**Figure 1:**
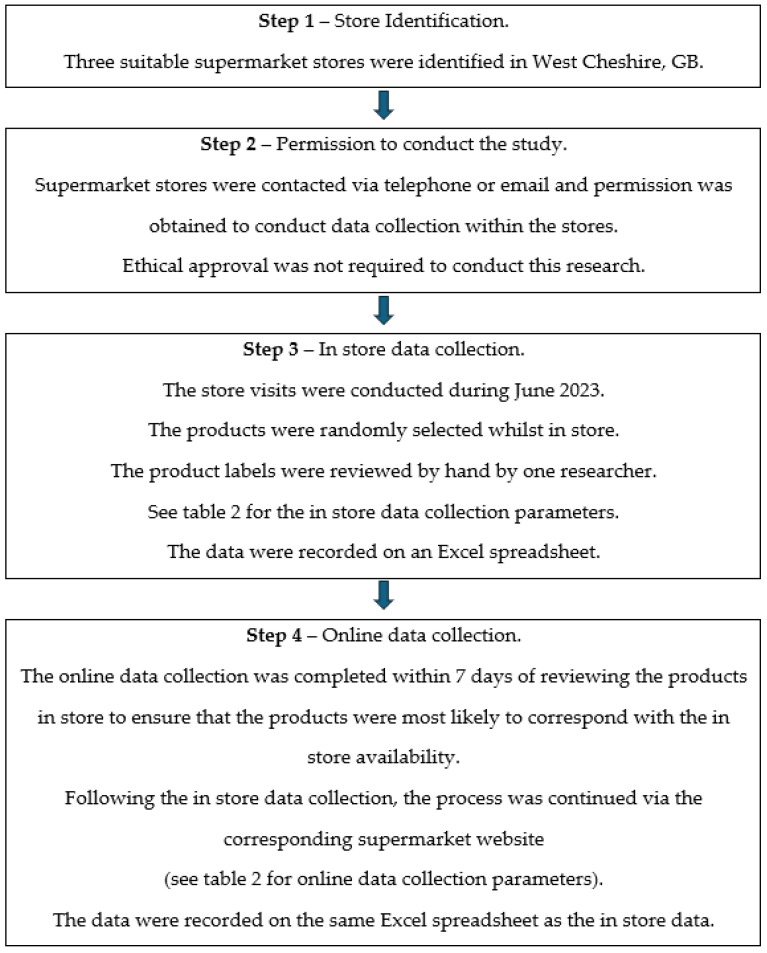
Flow chart to summarise the data collection process.

Throughout June 2023, 440 products were reviewed across the three supermarket stores, of which 406 were also available online via the corresponding supermarket’s websites. In 2022, the penetration rate for online food and grocery shopping was 11.6% [31], with 58% of 27 to 42 year olds opting to purchase via this route [32]. Therefore, it is an important platform for providing consumer information. The supermarket’s online consumer information is accessible to consumers on a national basis, providing a universal perspective on the information currently presented to consumers. When distance selling, Article 14 of Regulation (EU) No 1169/2011 [33] stipulates that retailers are obligated to provide mandatory consumer information, and it should reflect the information supplied when buying products in a physical environment. Therefore, supermarket websites should provide this information for consumers who complete their grocery shopping online. Any additional relevant information, for example, HCs and any supporting information should comply with the NHCR [2,34]. Figure 1 summaries the in-store and online data collection process. The products were selected in store by seeking the appropriate food category section within the store. The products were randomly selected from the shelf and reviewed by hand whilst in the store. Table 2 shows the data collection parameters that were used. In store, the product labels were reviewed to determine the presence of HCs, whereas online, the product information available on the website was used to identify HCs. The availability of product label images was variable online and was not always present in full to review. For example, it was common that only a pack shot of the front of the label was available but full product information was supplied as text on the website. This was used to assess online products. 

### 2.3. Health Claim Compliance Assessment

**Figure 2:**
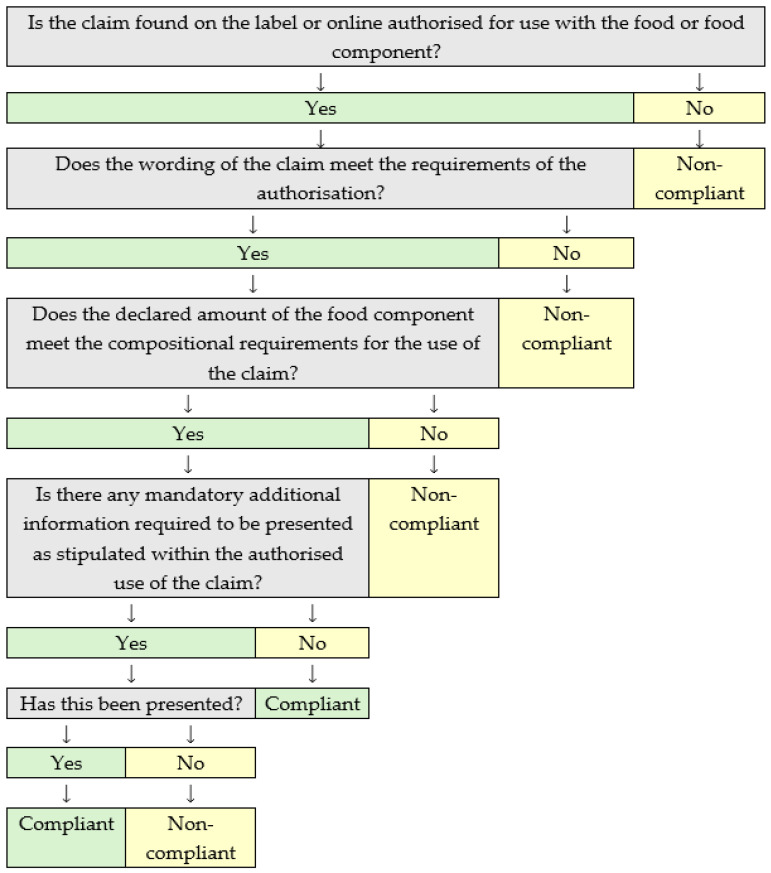
Health claim compliance assessment tool.

HC compliance was assessed using the parameters in Table 3, Figure 2, the NHCR [2] and the GBNHCR [6]. The use of the HC compliance assessment tool (see Figure 2) ensured that each of the HCs was subjected to a consistent assessment approach. The compliance of the HCs was examined by one researcher, Emma Coates (Registered Dietitian, MSc Food Regulatory Affairs), whilst under the supervision of two senior regulatory academic professionals.

### 2.4. Statistical Analysis

The data were analysed via SPSS Statistics (Version 28.0). Prior to the SPSS analysis, the data collected in store and online were coded to ensure they could be analysed using SPSS. Descriptive statistics were used to examine HC and FC frequencies for products in both settings and within each food category. A Chi-square test was used to analyse the statistical difference between in-store and online HC compliance. A one-way ANOVA followed by a Tukey HSD test was employed to determine any statistical differences in HC compliance between food categories. The data were assumed to be normally distributed, and *p*-values of <0.05 were considered to be statistically significant.

## 3. Results

### 3.1. In-Store and Online Health Claim Prevalence

A total of 440 products were reviewed in store, of which 406 were also available to review online via the corresponding retailers’ websites. In store, 26.3% of products carried an HC, and there was a slightly higher prevalence online (28.3%). Despite the lower number of products available online, there was an apparently slightly higher number of HCs in use online (*n* = 241) compared with in store (*n* = 231), which was not significantly different (*p* = 0.245). The distribution of products between the three food categories differed slightly, and this was due to the varying in-store ranges of products which were available within each category. D+DAs were the largest category in both settings, followed by FJ,FJD+FS, with T+Is making up the smallest category (see Table 4).

Although D+DAs were the largest category, FJ,FJD+FS carried the most HCs in store (43.3%). However, online, more HCs were found in D+DAs (42.7%). See Table 5, which shows the total number of HCs found on the products that were available in store and online.

### 3.2. Types of Health Claims

This study identified 30 different types of HCs in use. HCs referring to immune system health were the most prevalent, both in store (26.8%) and online (26.6%). Energy yielding and reductions in tiredness- and fatigue-related HCs were also common in both environments. Normal growth and development of bones were the most frequently used child-health-related HC in both settings (see Table 6).

#### Types of Health Claims Per Food Category

**Figure 3:**
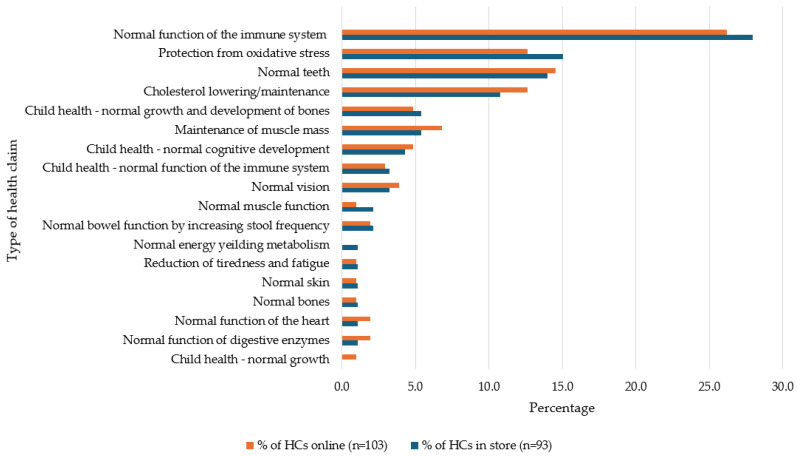
Percentage of types of health claims used on dairy and dairy alternative products available in store and online.

**Figure 4:**
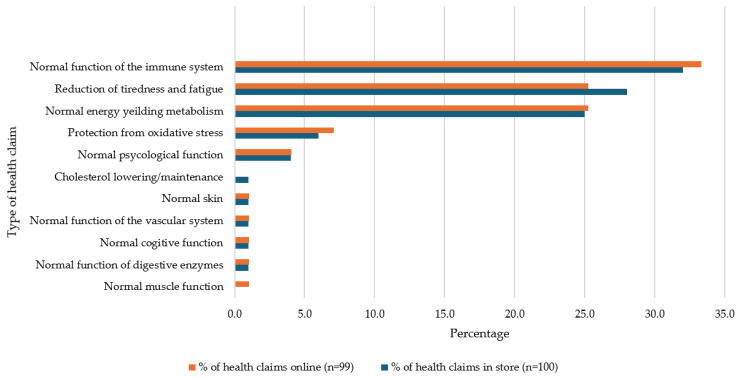
Percentage of types of health claims used on fruit juice, fruit juice drink and fruit smoothie products available in store and online.

**Figure 5:**
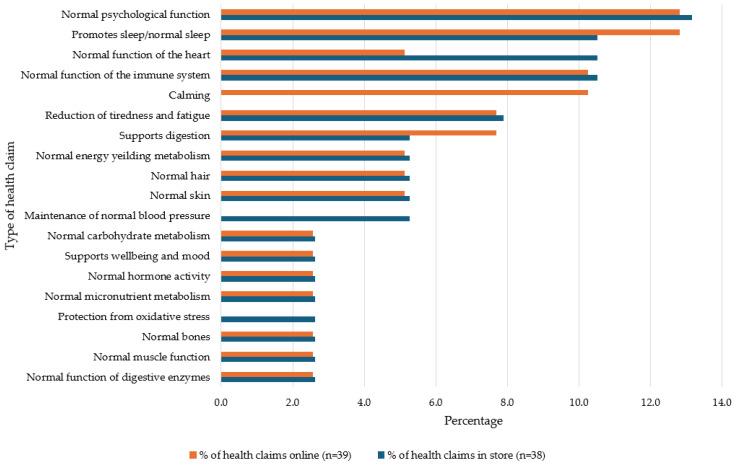
Percentage of types of health claims used on tea and infusion products available in store and online.

Within D+DAs, immunity-related HCs accounted for 27.9% of in-store and 26.2% of online HCs (see Figure 3). Maintenance of muscle mass contributed to over 10% of the HCs in this category across both settings. Regarding FJ,FJD+FS, the prevalence of immunity HCs was at its highest, at 32% in store and 33.3% online. Also, in this category, 25% of HCs were related to normal energy metabolism and a similar amount referring to reductions in tiredness and fatigue (see Figure 4). T+Is had the lowest prevalence of immunity-related HCs, 10.5% in store and 10.2% online. Normal psychological function HCs were the most prevalent in this category (see Figure 5).

### 3.3. Types of Food Components Used in Health Claims

**Figure 6:**
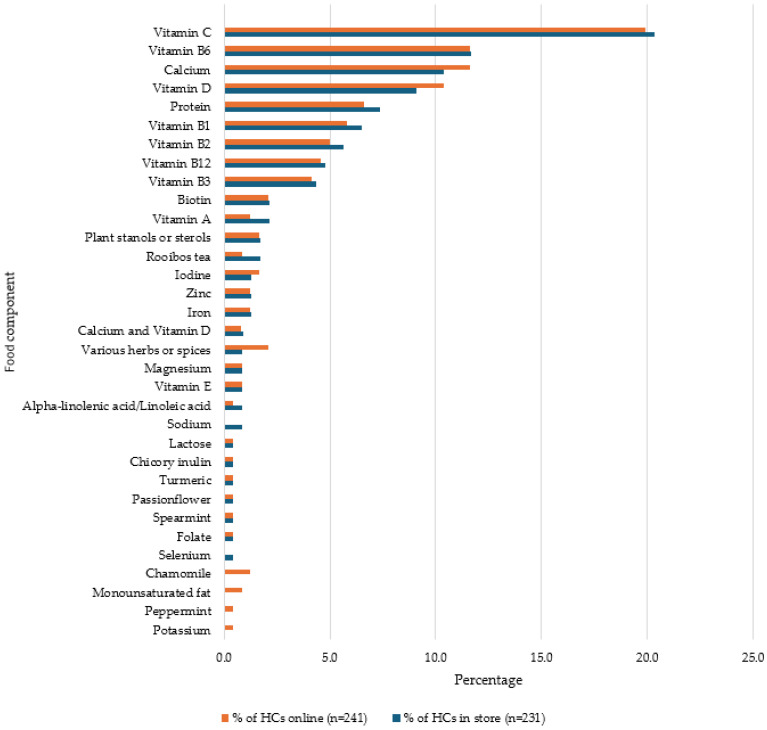
Percentage of types of food components used in health claims on products available in store and online.

During this study, 33 food components (FCs) were identified. Vitamin C was the most frequently used FC in store (20.3%) and online (19.9%). Calcium and vitamin B6 were also prevalent in both environments (see Figure 6). Where a combination of nutrients is included, for example, calcium and vitamin D, this refers to nutrients, as there are authorised HCs which refer to both, e.g., calcium and vitamin D are needed for the normal growth and development of bone in children. Therefore, the combination of the two nutrients was recorded as this reflected the actual HC used on the product.

### 3.4. Health Claim Compliance

HC compliance was higher in store (94.3%) than online (90.0%), albeit that this was not statistically different (*p* = 0.724) (see Table 7).

The HCs within the FJ,FJD+FS category were 100% compliant in both settings. However, T+Is contained the lowest number of compliant HCs, 76.3% in store and 64.1% online. In store, there was no statistically significant difference in HC compliance between D+DAs and FJ,FJD+FS (*p* = 0.092). This was mirrored online between the same groups (*p* = 0.129). There were statistically significant differences in HC compliance between D+DAs and T+Is in store (*p* = 0.009) and online (*p* < 0.001). Likewise, there were statistically significant differences between FJ,FJD+FS and T+Is in store (*p* < 0.001) and online (*p* < 0.001). Differences were calculated using a one-way ANOVA followed by a Tukey post hoc test.

The basis for non-compliance varied within this study. Non-compliant wording of HCs (*n* = 8 in store and online) and nutritional composition (*n* = 1 in store and online) occurred within the D+DAs category, whereas use of unauthorised HCs was observed in both D+DAs (*n* = 1 in store and online) and T+Is (*n* = 9 in store and *n* = 14 online). The unauthorised HCs within T+Is were related to botanical ingredients and not present on the GBNHCR; therefore, they were deemed unauthorised.

## 4. Discussion

This study evaluated the prevalence and compliance of HCs used on prepacked foods sold within three GB supermarkets. The prevalence of HCs was higher when compared with data from 2016 [19]. There was a slightly higher number of products in store carrying an HC 26.3% compared with 28.3% online. HC compliance was good, both in store (94.3%) and online (90.0%). There was no difference in overall HC compliance between in-store and online products, and the HC violations observed were due to non-compliant wording of HCs or use of non-authorised HCs relating to botanical ingredients.

The prevalence of HCs found within this study differs to data from the Irish supermarket-based studies by Lalor et al. (2009) [18] and Offe et al. (2022) [17]. Overall, in the current study, 26.3% of products reviewed in store carried an HC, which is substantially higher than Lalor et al. [18] (17.8%) and Offe et al. [17] (10.5%). The UK prevalence of HCs within the multicentred European study by Hieke et al. (2016) [19] was also lower (10.9%) than the data obtained in this study. This may be a result of the different structure of the food categories included with the studies and the time that has elapsed since the studies were conducted. The current study included D+DAs as a food category. Both milk-based and dairy alternative products were included. The studies by Lalor et al. (2009) [18], Offe et al. (2022) [17] and Hieke et al. (2016) [19] included dairy products but not dairy alternatives. Within Europe, sales of dairy alternatives, such as plant-based milk drinks, cheese and yogurt alternatives, have increased by 10% over the past decade and are projected to increase further by 2025 [35]. The number of food products included in the current study is comparatively small compared to the other studies; therefore, it is challenging to make general conclusions regarding the current HC prevalence in GB.

As concerns dairy products, the data from this study suggest that there may a similar prevalence to that documented by Lalor et al. (2009) [18]. In store, 40.3% of D+DAs in this study carried an HC. Lalor et al. (2009) [18] also found that the prevalence of HC use on dairy products was relatively high and was reported as milk (36%), butter/spreads (30%), yogurts/yogurt drinks (50%) and cheese (16%). Conversely, Offe et al. (2022) [17] reported a lower prevalence across the dairy foods included in that study. Only yogurts/yogurt drinks and cheese were evaluated, and there was a decrease in HC prevalence when compared with the earlier data presented by Lalor et al. [18] in 2009. The decrease was attributed to the implementation of the NHCR, which occurred between the studies being conducted [17]. Hieke et al. (2016) [19] also evaluated dairy products. The data were provided per overall product group, as opposed to individual foods. Per country data were not provided, but, overall, 13% of dairy products within that study carried an HC.

Only Lalor et al. (2009) [17] included tea products as a specific food category. Hieke et al. (2016) [19] also evaluated them but as part of a broader category. ‘Beverages’ were reviewed, which included teas. Other products, such as waters, cordials, and electrolyte drinks, were also included. Specific data on teas were not provided. The in-store data from the current study show a lower prevalence of HC on T+Is when compared with Lalor et al. (2009) [18], 16.4% versus 24%. A smaller number of tea products was included in the Lalor et al. [18] study (*n* = 38), which is much lower than the volume included in this study (*n* = 104 in store). This may account for the difference in prevalence, in addition to the impact of the NHCR since the Lalor et al. study (2009) [18] was published. Looking at FJ,FJD+FS, the in-store prevalence of HCs in this category was higher than the data presented by Lalor et al. (2009) [18] and Offe et al. (2022) [17] by 17.5 percentage points. This may be reflective of evolving trends in the types of HCs in use.

This study identified 30 different HCs in use across three food categories. Immunity-related HCs were the most prevalent overall, at 26.8% in store and 26.6% online. When considering the in-store data, the majority of these types of HCs were present in the FJ,FJD+FS (32%) and D+DAs (26.2%) categories. Immunity HCs were also prevalent in the Lalor et al. (2009) [18] study, where 15.9% of yogurts and yogurt drinks and 40% of fruit juices and fruit smoothies carried this type of HC. Furthermore, Hieke et al. (2016) [19] reported that overall immunity HC prevalence was 8.8%. Information regarding HC type per food category was not provided in that study. Offe et al. (2022) [17] acknowledged that HCs on fruit juices and smoothies are commonly accepted, and the value consumers place on HCs can vary depending on the food category. The use of immunity-related HCs has been prevalent in this food category since the Lalor et al. (2009) [18] study was published. In addition to immune system health HCs, reductions in tiredness and fatigue, and normal energy-yielding metabolism HCs, were also prevalent in the data collected in this study across both settings (see Table 6). Neither Lalor et al. (2009) [18] or Offe et al. (2022) [17] discussed this type of HC; therefore, it is not possible to compare the data. However, Hieke et al. (2016) [19] reported that HCs relating to ‘energy and drive functions’ contributed to 4.4% of overall HCs.

During this study, 33 FCs were recorded, and, overall, vitamin C was the most frequently used FC in store (20.3%) and online (19.9%). Vitamin B6 was the second-most-frequent FC in use, present in 11.7% of HCs in store and 11.6% online. Lalor et al. (2009) [18] provided data regarding the prevalence of FCs in use with HCs; however, the data were grouped together as ‘vitamins’ or ‘minerals’ rather than individual nutrients, as per this study. When the data for vitamins and minerals are grouped together in this study, vitamin-related HCs account for 68.8% of HCs in store, whereas mineral-related HCs have a lower prevalence, 17.4% in store. This is a higher prevalence than reported by Lalor et al. (2009) [18], where 11.9% of HCs were vitamin-related and 10.1% included minerals. Offe et al. (2022) [17] did not provide data on the FCs in use with HCs. Conversely, Hieke et al. (2016) [19] presented data regarding some individual FCs, with vitamin C being present in 2% of the HCs reviewed in the study.

The compliance of HCs in accordance with the NHCR was investigated during this study. Overall compliance in both settings was good and marginally higher in store (94.3%) than online (90.0%), but this was not statistically significantly different (*p* = 0.245). There are no existing data to directly compare the combination of in-store and online data in this study. However, the in-store HC compliance for the product types included in the current study can be compared with existing data. In this study, the in-store HC compliance rate in the D+DA category was 95.7%. When compared with Bonaccorsi et al. (2018) [28], who reviewed food labels within Italian supermarkets, the compliance rate on DA+As products (including milk, soy products, fermented milk and yogurt) collectively was 84.5%. Whilst this demonstrates a lower compliance rate, the number of D+DAs reviewed by Bonaccorsi et al. (2018) [28] was very limited when compared with this study: *n* = 25 versus *n* = 176. When reviewing the HC compliance rates on T+Is, Kerrigan (2020) [20] found a much higher rate of HC non-compliance when compared with the data from this study. Kerrigan (2020) [20] reported that 100% of HCs used on 107 herbal and green products reviewed in Irish health food shops were unauthorised and, therefore, non-compliant. Whilst compliance was lowest in the T+I category within this study, it was not as severe as the findings from Kerrigan (2020) [20], with 76.3% of in-store HCs being compliant across 124 products. Although the food category is not comparable with those included in the current study, Conway et al. (2023) [27] investigated the HC compliance on infant formulas for sale over the counter in GB. The study found that 18% of all infant formulas included in the evaluation carried HCs that could be considered to be non-permitted as they were not included in the GBNHCR [6]. Within the Conway et al. (2023) [27] study, the data were collected both in store and online; however, they were analysed as a whole, and no differentiation between the in-store and online HC compliance was presented. The lower online compliance rate within the current study is consistent with Dominguez et al. (2021) [21]. Although the product type is not directly comparable with those included in the current study, when evaluating the compliance of HCs on food supplements for pregnant women, Dominguez et al. (2021) [21] reported 100% compliance on products bought in store but 85.7% compliance on products purchased online. No statistical analysis was performed by Dominguez et al. as part of the study. In 2022, the Danish Veterinary and Food Administration (DVFA) also reported increased use of claims via digital platforms; however, no specific data were presented regarding the compliance rates between in-store and online settings [36]. Again, whilst the product type is not directly comparable with those included in the present study, Molina-Juan et al. (2021) [24] found that only 25% of the HCs in use on creatine monohydrate-containing sports supplements (CMCSSs) were compliant with the NHCR. The study reviewed the HCs that were present on 167 CMCSSs available via online shopping platforms, Amazon and Google Shopping websites. The substantially lower HC compliance rate in the Molina-Juan et al. (2021) [24] study may be related to the type of product investigated, in that sports supplements may not be regulated as well as common groceries, such as those included in the current study. Further, poor online HC compliance on sport supplement products was documented by Estevan-Navarro et al. (2021) [23], where caffeine HCs on sport supplement labelling were investigated. Estevan-Navarro et al. (2021) [23] reviewed 42 caffeine supplements, which were available via the same online platforms as those used in the Molina-Juan et al. (2021) [24] study. All of the products carried a caffeine-related HC, none of which were authorised. Alternatively, the lower HC compliance demonstrated in these studies may be a result of poorer regulation of HCs in use via online shopping platforms that are not online supermarket shopping websites. Further research is needed to develop more insight into this.

The use of non-authorised HCs observed within T+Is is somewhat consistent with the products evaluated by Kerrigan (2020) [20]. Similar violations were observed on infant formulas, which were investigated in studies by Conway et al. (2023) and on meal replacement bars by Vivante et al. (2022) [25]. Bonaccorsi et al. (2018) [28] reported non-compliance due to a lack of mandatory supporting HC information, which was not observed in the current study. Although the food categories are not directly compatible with those included in the present study, Wicklow et al. (2014) [26] also documented this violation, in addition to exaggeration of the health benefits of a product when evaluating HCs used on paediatric food supplements. Whilst HCs may be used by consumers to identify and make healthier food choices [37,38,39], there are also data to suggest that HCs can be a hindrance to some consumers. They may lead to confusion, or they are a distraction from other relevant food information when making decisions about the foods they buy; for other consumers, they do not offer very much assistance at all [40,41,42,43]. Nevertheless, non-compliant HCs are misleading to any consumer and are in breach of the NHRC [2].

The strengths of this study are that the range of products included in the study is accessible to consumers on a national basis, both in store and online. The use of three of the major GB grocery retailers provided this insight and aligns to the methodology applied by Offe et al. (2022) [17]. Whilst the in-store data collection was conducted in one small region of GB, national retailers were included in the study, which sell the same products across the country. Likewise, the corresponding retailer websites are reflective of the national access to the products on sale and the HC date presented with them. The additional evaluation of online HC information further galvanises the national reach of the data. Therefore, the results of this study are a representation of the national status of HC compliance within the food categories and by the retailers included. A range of products were reviewed across three different food categories, and both the prevalence and compliance of the HCs within them were evaluated, which is unique within the current pool of existing data. The compliance data were subjected to statistical analysis, which was missing in previous studies. The random selection of products whilst in store permitted a non-biased approach to reviewing the HC information, and the use of standardised methodology throughout the study was engaged to ensure consistency across all retailers and food categories, preventing bias where possible. One researcher conducted the data collection in the current study; therefore, there was no differentiation in the collection and evaluation of the data. The study size (*n* = 440) within this study may appear relatively small when compared with other studies by Lalor et al. (2009) [18]—*n* = 1880, Hieke et al. (2016) [19]—*n* = 2034 and Offe et al. (2022) [17]—*n* = 1636. However, the sample size included in this study is larger than the overall sample size for the UK within the Hieke et al. (2016) [19] study—*n* = 368 (UK sample size), and when products from comparable food categories are taken into consideration, Offe et al. (2022) [17]—*n* = 398. However, the sample size of products from comparable food categories within the Lalor et al. (2009) [18] study was larger, *n* = 654. The data from this study may benefit from extended research to increase their power; however, at present, they are comparable with currently available data.

There were limitations within this study. There are several other GB grocery retailers, which were not included due to limited time and resources, or permission was not granted to conduct in-store data collection. This excluded a wider range of products and HC information that GB consumers also access. Similarly, alternative online shopping channels, such as Amazon UK, Ocado or Google Shopping websites, were not included. These platforms sell a wide range of grocery products, which were not evaluated. Independent retailers were also excluded from this study, which may highlight differences in the prevalence and compliance of HCs in use. The data collection for each store and website included in this study was conducted on different dates. Therefore, the data collected on each different day were a snapshot of the products that were available on that day. Ongoing data collection may highlight changes in HC information as products are introduced or existing products are reformulated. This may have an impact on the rates of compliance over time. Fewer food categories were included in this study when compared with the studies by Lalor et al. (2009) [18], Hieke et al. (2016) [19] and Offe et al. (2022) [17]. This limits the opportunity to fully evaluate HC compliance across other common food categories and does not permit further HC compliance comparison with those studies. The in-store data collection would have been more accurate if two researchers conducted the study independently, and photos of product labels were taken for later traceability. A third researcher may have been beneficial should any disagreement between the two researchers have arisen.

Whilst this study offers a good insight into the current prevalence and compliance of HCs within GB supermarkets, further research is required to investigate more physical and digital locations where this information is presented to consumers. Additional product categories such as breakfast cereals and previously less investigated categories such as plant-based alternatives should be reviewed. Although the compliance rates within this study are better than comparable European studies, the UK government recently proposed changes to the enforcement of retained NHCR [44]. The reforms include the introduction of an improvement notices regime [44], which is not currently in effect. The aim of the new regimen is to allow for early enforcement authority intervention where businesses are in breach of the NHCR. It is anticipated that this will prevent costly legal proceedings and facilitate a more efficient way to address non-compliance [44]. The consultation on these changes was open until 31st October 2023, and the outcomes are awaited.

## 5. Conclusions

This study investigated the prevalence and compliance of HCs within GB and highlighted a number of differences when compared with the limited existing data. Overall, in-store HC prevalence has increased in the past 14 years, in addition to increases in HC use on FJ,FDJ+FS. However, there is a decrease in HCs used on T+Is. The prevalence of HC relating to immune system health has increased, in addition to increases in HCs related to reductions in tiredness and fatigue and normal energy-yielding metabolism. This aligns with an increased prevalence of HCs that refer to vitamins and minerals. The compliance of HCs on products available via GB supermarkets is generally good, with no statistically significant differences between in-store and online compliance. Despite the decrease in HCs in use on T+Is, this was the least compliant category within this study. The HC violations observed in the present study were predominantly due to non-compliant wording of HCs and nutritional composition or the use of non-authorised HCs relating to botanical ingredients, which is consistent with data from other studies [20,21,22,23,24,25,27,28].

The data from this study could help policymakers to benchmark HC compliance within GB and could support the development of strategies to provide guidance on the compliant use of HCs, monitor HC compliance and enforce the NHCR [2] more effectively. A recent consultation on enforcement by the UK government proposed more proactive early intervention in the management of non-compliance. The outcomes and potential impact of these proposals are yet to be seen. However, it may prove to be constructive for food manufacturers and retailers in improving the compliance of any HCs they use. Standardised and unambiguous guidelines for managing non-compliance could support food manufacturers and retailers to use HCs in the most appropriate and authorised manner. Ultimately, improved HC compliance benefits the consumer and offers reassurance that the products they consume are safe and have scientifically substantiated HCs that they can trust. This allows the consumer to make informed decisions when they are buying food and to include foods that may bring particular health benefits that could have a positive impact on their overall health and well-being.

## Figures and Tables

**Table 1:** Summary of product types per food category.

Dairy and Dairy Alternatives	Fruit Juices, Fruit Juice and Fruit Smoothies	Teas and Infusions
**Milk-containing dairy**	Ambient fruit juice	Black tea
Ambient milk (long life milk)	Chilled fruit juice	Black tea decaffeinated
Chilled fresh milk	Ambient fruit juice drinks	Green tea
Ambient lactose free milk	Chilled fruit juice drinks	Green tea decaffeinated
Chilled lactose free milk	Ambient fruit smoothies	Rooibos tea
Powdered milk	Chilled fruit smoothies	Herbal/fruit tea/infusion
Milkshake/milkshake drink		Instant tea
Cream		White tea
Cheese		
Yogurt		
Yogurt drink		
**Plant based dairy alternatives**		
Ambient milk alternative		
Chilled milk alternative		
Milkshake/milkshake drink alternative		
Cream alternative		
Cheese alternative		
Yogurt alternative		
Yogurt drink alternative		
Spread alternative		

**Table 2:** Data collection parameters.

**In store data collection**
Product number Store name/code Product name Brand Study food category Health claim present (Yes/No) If yes, number of health claims present Type of claim * Wording of claim Additional information or warnings on pack Name of food component that the claim refers to, e.g., vitamin C, calcium Amount of the nutrient per 100 g/mL in mg, if required % RI per 100 mL/g, if required % energy, if required
**Data collection continued online**
Is the product available on the supermarket’s website? (Yes/No) Health claim present? (Yes/No) If yes, number of health claims present Type of claim * Wording of claim Additional information or warnings on pack

* standardised categories were created to ensure consistency in recording type of health claim (see Table 6).

**Table 3:** Health claim compliance parameters.

The health claim	Type of claim Wording of claim
Nutrient, ingredient or component	Name of food component that the claim refers to, e.g., vitamin C, calcium
Compositional requirements	Amount of the food component per 100 g/mL, if required % RI per 100 mL/g, if required
Supporting information	Additional mandatory or relevant information or warnings provided, if required

**Table 4:** Number of products within each food category on products available in store and online.

Food Category	Number Products Reviewed in Store (*n* = 440)	Of Which Available Online (*n* = 406)
Dairy and dairy alternatives	176	170
Fruit juices, fruit juice drinks and fruit smoothies	140	128
Teas and infusions	124	108

**Table 5:** Prevalence of health claims within each food category found on products available in store and online.

Food Category	Total Number of Health Claims Found on Products Available in Store (*n* = 231)	% of Overall in Store Health Claims	Total Number of Health Claims Found on Products Available Online (*n* = 241)	% of Overall Online Health Claims
Dairy and dairy alternatives	93	40.3%	103	42.7%
Fruit juices, fruit juice drinks and fruit smoothies	100	43.3%	99	41.1%
Teas and infusions	38	16.4%	39	16.2%

**Table 6:** Types of health claims in use on overall products available in store and online.

Type of Claim	Number of Health Claims in Store	% of Total Health Claims (*n* = 231)	Number of Health Claims Online	% of Total Health Claims (*n* = 241)
Normal function of the immune system	62	26.8	64	26.6
Reduction of tiredness and fatigue	33	14.3	29	12.0
Normal energy-yielding metabolism	27	11.7	28	11.6
Normal bones	14	6.1	16	6.6
Child health—normal growth and development of bones	14	6.1	13	5.4
Maintenance of muscle mass	10	4.3	13	5.4
Normal psychological function	9	3.9	9	3.7
Protection from oxidative stress	8	3.5	8	3.3
Normal function of digestive enzymes	7	3.0	7	2.9
Cholesterol lowering/maintenance	6	0.3	7	2.9
Normal skin	6	2.6	6	2.5
Normal function of the heart	5	2.2	3	1.2
Normal teeth	4	1.7	5	2.1
Promotes sleep/normal sleep	4	1.7	4	1.7
Child health—normal cognitive development	3	1.3	4	1.7
Normal muscle function	2	0.9	4	1.7
Supports digestion	2	0.9	4	1.7
Calming	0	0.0	4	1.7
Normal hair	2	0.9	2	0.8
Child health—normal function of the immune system	2	0.9	2	0.8
Maintenance of normal blood pressure	2	0.9	0	0.0
Child health—normal growth	1	0.4	2	0.8
Normal hormone activity	1	0.4	2	0.8
Normal vision	1	0.4	1	0.4
Normal cognitive function	1	0.4	1	0.4
Normal function of the vascular system	1	0.4	1	0.4
Normal carbohydrate metabolism	1	0.4	1	0.4
Supports wellbeing and mood	1	0.4	0	0.0
Normal bowel function by increasing stool frequency	1	0.4	0	0.0

**Table 7:** Compliance of health claims found on products available in store and online.

	Products Reviewed in Store	Of Which Available Online	*p* Value *
Total number of health claims	231	241	
Number of compliant health claims	218	217	
Overall health claim compliance	94.3%	90.0%	0.724

* Statistically significant difference = *p* < 0.05. Differences were calculated using a Chi-square test.

## Data Availability

The original contributions presented in the study are included in the article, further inquiries can be directed to the corresponding author.
